# Comparative Effectiveness of Angiotensin-Converting Enzyme Inhibitors and Angiotensin II Receptor Blockers in Terms of Major Cardiovascular Disease Outcomes in Elderly Patients: A Nationwide Population-Based Cohort Study

**DOI:** 10.1097/MD.0000000000001751

**Published:** 2015-10-30

**Authors:** Shu-Chen Chien, Shuo-Ming Ou, Chia-Jen Shih, Pei-Wen Chao, Szu-Yuan Li, Yi-Jung Lee, Shu-Chen Kuo, Shuu-Jiun Wang, Tzeng-Ji Chen, Der-Cherng Tarng, Hsi Chu, Yung-Tai Chen

**Affiliations:** From the School of Pharmacy, College of Pharmacy, Taipei Medical University (S-CC), Department of Pharmacy, Taipei Medical University Hospital (S-CC), Clinical Research Center, Taipei Medical University Hospital (S-CC), School of Medicine, National Yang-Ming University (S-MO, C-JS, S-YL, Y-JL, S-CK, S-JW, D-CT, Y-TC), Division of Nephrology, Department of Medicine, Taipei Veterans General Hospital (S-MO, S-YL, D-CT), Institute of Clinical Medicine, National Yang-Ming University, Taipei (S-MO, D-CT), Department of Medicine, Taipei Veterans General Hospital, Yuanshan Branch, Yilan (C-JS), School of Medicine, Taipei Medical University (P-WC), Department of Anesthesiology, Wan Fang Hospital, Taipei Medical University (P-WC), Department of Neurology, Taipei City Hospital, Ren Ai Branch, Taipei (Y-JL), National Institute of Infectious Diseases and Vaccinology, National Health Research Institutes, Miaoli County (S-CK), Division of Infectious Diseases, Taipei Veterans General Hospital (S-CK), Institute of Brain Science, National Yang-Ming University (S-JW), Department of Neurology, Neurological Institute, Taipei Veterans General Hospital (S-JW), Department of Family Medicine, Taipei Veterans General Hospital (T-JC), Department and Institute of Physiology, National Yang-Ming University (D-CT), Department of Chest, Taipei City Hospital, Heping Fuyou Branch (HC); and Division of Nephrology, Department of Medicine, Taipei City Hospital, Heping Fuyou Branch, Taipei, Taiwan (Y-TC).

## Abstract

Renin and aldosterone activity levels are low in elderly patients, raising concerns about the benefits and risks of angiotensin-converting-enzyme inhibitors (ACEIs) and angiotensin II receptor blockers (ARB) use. However, data from direct comparisons of the effects of ACEIs on ARBs in the elderly population remain inconclusive.

In this nationwide study, all patients aged ≥ 70 years were retrieved from the Taiwan National Health Insurance database for the period 2000 to 2009 and were followed up until the end of 2010. The ARB cohort (12,347 patients who continuously used ARBs for ≥ 90 days) was matched to ACEI cohort using high-dimensional propensity score (hdPS). Intention-to-treat (ITT) and as-treated (AT) analyses were conducted.

In the ITT analysis, after considering death as a competing risk, the ACEI cohort had similar risks of myocardial infarction (hazard ratio [HR] 0.92, 95% confidence interval [CI] 0.79–1.06), ischemic stroke (HR 0.98, 95% CI 0.90–1.07), and heart failure (HR 0.93, 95% CI 0.83–1.04) compared with the ARB cohort. No difference in adverse effects, such as acute kidney injury (HR 0.99, 95% CI 0.89–1.09) and hyperkalemia (HR 1.02, 95% CI 0.87–1.20), was observed between cohorts. AT analysis produced similar results to those of ITT analysis. We were unable to demonstrate a survival difference between cohorts (HR 1.03, 95% CI 0.88–1.21) after considering drug discontinuation as a competing risk in AT analysis.

Our study supports the notion that ACEI and ARB users have similar risks of major adverse cardiovascular events (MACE), even in elderly populations.

## Abstract 2

Supplemental Digital Content is available in the text

## INTRODUCTION

The prevalence of hypertension increases dramatically with advanced age and results in considerable cardiovascular morbidity and mortality.^1,2^ The benefits from antihypertensive therapy in elderly patients that can be expected to depend primarily on the effect of reducing cardiovascular complications as well as the drug tolerability and safety.^3–6^ A meta-analysis of 31 trials with 190,606 participants demonstrated similar blood pressure control among different classes of antihypertensive drugs, even in the elderly population.^7^ Results of previous randomized clinical trials showed angiotensin-converting enzyme inhibitors (ACEIs) or angiotensin II receptor blockers (ARBs) exert cardiovascular protective effects compared to placebo or other active treatment.^8–13^ Up to date, however, comprehensive head-to-head randomized studies specifically comparing the efficacy of ACEIs versus ARBs in elderly patients have been rarely performed. The potential benefits of renin–angiotensin–aldosterone system (RAAS) blockers in elderly patients must be weighed against the potential risks of acute kidney injury and hyperkalemia due to age-related reductions in serum renin and aldosterone levels.^14^

Two randomized clinical trials (RCTs) demonstrated that ACEIs and ARBs were equally effective in reducing blood pressure in elderly patients with hypertension.^15,16^ Although both treatments can achieve similar blood pressure control, the Evaluation of Losartan in the Elderly (ELITE) Study and the ELITE II Study produced inconclusive results concerning cardiovascular benefits of ACEIs versus ARBs in elderly patients with heart failure.^17,18^ Similarly, previous observational studies have produced conflicting results regarding which RAAS blockers favor clinical outcomes in elderly patients.^19,20^ These observational studies may be limited due to small samples, short follow-up periods, and lack of considering the impact of death and drug adherence in their analyses. The competing risk of death in elderly patients may be especially high because of multiple coexisting chronic diseases. Drug adherence to ACEIs in elderly hypertensive patients may also be difficult to achieve as this population is usually complicated by occurrence of side effects such as dry cough. Therefore, traditional statistical method in previous observational studies can overestimate the risk of disease by failing to account for the competing risk of death or drug discontinuation.

Given the lack of sufficient clinical trial and observational data, we conducted a high-dimensional propensity score (hdPS)-matching study and considered death and drug adherence as competing risks in the assessment of the effects of ACEI- and ARB-based treatment strategies on long-term mortality, major adverse cardiovascular events (MACE), and renal outcomes in patients aged ≥ 70 years in Taiwan between 2000 and 2010.

## METHODS

### Data Source

This study used data from Taiwan's National Health Insurance Research Database (NHIRD). Taiwan's National Health Insurance (NHI) program, launched in 1995, is a universal, state-operated health program that covers approximately 99% of Taiwan's population. In 1999, the Bureau of the NHI began to release all claims data after encryption of all personal information to the public for scientific research purposes. Multiple deidentified NHI databases, including NHI enrollment files, claims data, detailed orders, and drug prescriptions (including data for hospital inpatient and outpatient care, emergency room services, dental services, and traditional Chinese medicine care), are available to researchers. Several published studies addressing the effects of RAAS blockers have also been based on the NHIRD.^21–24^ Disease diagnoses were defined based on medical claims using International Classification of Diseases, Ninth Revision, Clinical Modification (ICD-9-CM) diagnostic codes.

### Ethical Approval

Due to the retrospective nature of this study with deidentified secondary data, it was exempt from full review by the Institutional Review Board.

### Study Design

This nationwide population-based cohort study compared the effects of ACEIs and ARBs on all-cause mortality and MACE in elderly patients. We extracted data from all subjects aged ≥ 70 years with hypertension, including demographic variables, diagnosis and procedure codes, and information about outpatient visits, hospital admissions, and drug prescriptions, for the period of January 2000 to December 2009. Patients with chronic (continuous for ≥ 90 days) use of any ACEI or ARB were included (Supplementary Figure 1, http://links.lww.com/MD/A473). The index date was defined as the day after 90 days of continuous use. We also extracted data from the period of January 1995 to December 1999 to define comorbidities. We excluded patients who used the opposite drug before the index date; were receiving dialysis; were hospitalized within 90 days before the index date; had histories of cerebrovascular disease, myocardial infarction, or end-stage renal disease; or were kidney transplant recipients.

### Outcomes

The primary outcomes were all-cause mortality, hospitalization with the principal diagnosis of heart failure (ICD-9-CM code 428.x), and MACE, including hospitalization with the principal diagnosis of ischemic stroke (ICD-9-CM code 433.x, 434.x, or 436) and myocardial infarction (MI; ICD-9-CM code 410.x). The secondary outcomes were hospitalization with the principal diagnosis of acute kidney injury (ICD-9-CM code 584.x) and hyperkalemia (ICD-9-CM code 276.7). All subjects were enrolled between January 1, 2000 and December 31, 2009 and followed until death or December 31, 2010.

### Baseline Characteristics

We examined the baseline sociodemographic characteristics of the elderly cohort, including age, sex, monthly income (New Taiwan dollars [NT$] < 19,100, NT$ 19,100–42,000, and NT$ >42,000), urbanization level, and Charlson Comorbidity Index (CCI) score.^25^ Urbanization levels in Taiwan are divided into 4 strata according to the Taiwan National Health Research Institute, in which level 1 is referred to as the “most urbanized” and level 4 as the “least urbanized.” The CCI score is used widely to determine overall systemic health, with each score increase reflecting a stepwise increase in cumulative mortality.^25^

We also examined other systemic diseases and risk factors for cardiovascular disease not included in the CCI score, including hypertension, coronary artery disease, dyslipidemia, arrhythmia, preexisting valvular heart disease, and drug abuse. Concomitant medications associated with cardiovascular indication implying associated cardiac diseases were also taken into consideration; these included alpha, beta, and calcium-channel blockers, diuretics, other anti-hypertensive drugs, antiplatelet agents, warfarin, nitrate, statins, dipyridamole, steroids, estrogen or progesterone, nonsteroidal antiinflammatory drugs, proton-pump inhibitors, and antihyperglycemic drugs.

### ACEI/ARB Exposure

For each pharmacy record of ACEI or ARB prescription, we identified the drug type and dose, dispensing date, and prescribed duration. The drug continuation was defined as ACEI/ARB prescription with a subsequent prescription within 90 days.^26^ On the other way, drug discontinuation was defined as more than 90 days of exhausting the drug supply for the prior prescription. The drug exposure period was defined as time duration extending from the beginning date of the exposure of each ACEI/ARB to the date of last dispensing.

### High-Dimensional Propensity Score Matching

Because clinical trials are traditionally expensive and enrollment of a sufficient number of patients can be difficult, large health care claims databases are frequently used to determine the effects of medication use on clinical outcomes. This approach may reflect real-world clinical practice, reveal rare drug effects, and avoid the delay of data collection. However, the use of claims databases in pharmacoepidemiology is associated with a primary concern regarding the incompleteness of information on potential confounders due to unmeasured frailty. The hdPS algorithm was proposed for use with such large health care claims databases to prioritize thousands of covariates at the demographic level, drug codes, and ordering of laboratory/diagnostic procedures based on variables’ potential to cause multiplicative bias in multistep processes.^27^ Similar to previous studies,^28,29^ the predefined covariate (Supplementary Table 1, http://links.lww.com/MD/A473) and the top 500 empirical covariates most likely to cause bias in the inclusion processes were selected (data not shown). Logistic regression was used to predict the probability of receiving ARBs and to calculate the hdPSs of all patients in the hdPS model. Using 1:1 nearest neighbor hdPS matching without replacement, 1 elderly patient receiving ACEIs was matched to each elderly patient receiving ARBs (caliper width = 0.019; 0.1 standard deviation of hdPS logit).

### Statistical Analysis

Descriptive statistics were used to characterize the study populations. Baseline characteristics were compared using the Pearson χ^2^ test for categorical variables and equivalence test for the mean differences between groups. hdPSs for the likelihood of using ARBs were calculated using a multistep process.^27^ The standardized difference was used to discern differences between matched groups. The incidence rates of MACE in the 2 groups were calculated using Poisson distribution. Intention-to-treat (ITT) and as-treated (AT) analyses were conducted. ITT analysis ignores noncompliance and drug switching or drug withdrawal after enrollment, which preserves baseline comparability and provides conservative estimates of differences between treatment groups. In AT analyses, elderly patients were censored on the day that they switched or discontinued ACEIs/ARBs. The cumulative incidence of serious cardiovascular events was estimated using the Kaplan–Meier method, and differences between cohorts were evaluated using the log-rank test. Cox regression models with a conditional approach and stratification were used to calculate hazard ratios (HRs) and 95% confidence intervals (CIs) for the occurrence of MACE in each group.^30^ Besides, the competing-risks regression by the method of Fine and Gray's model was also calculated.^31^ In this model, death and drug discontinuation were calculated as competing risks. The likelihood ratio test was used to examine interactions between the occurrence of serious cardiovascular events using ARBs and the following variables: age, sex, CCI score, diabetes mellitus, hypertension, chronic kidney disease, heart failure, myocardial infarction, coronary artery disease, and cerebrovascular disease. Subgroup analyses were also performed accordingly. Data linkage, processing, and analysis were conducted with the SQL Server 2012 (Microsoft Corporation, Redmond, WA). We used SAS version 9.3 (SAS Institute, Cary, NC) to calculate hdPSs and used STATA statistical software (version 13.0; StataCorp, College Station, TX) to perform other statistical analyses. The designated level of statistical significance was *P* < 0.05.

## RESULTS

### Characteristics of the Study Population

During the study period, a total of 31,506 ARB users and 47,646 ACEI users who met the inclusion criteria were enrolled in the study. Before matching, ARB users were older, had higher CCI scores, had more comorbid conditions, and received more concomitant medications compared with ACEI users; female sex was also more predominant in the ARB cohort. After hdPS matching, 12,347 ARB users and 12,347 ACEI users were included in analyses. Characteristics of the study population are detailed in Table 1 .

**TABLE 1:**
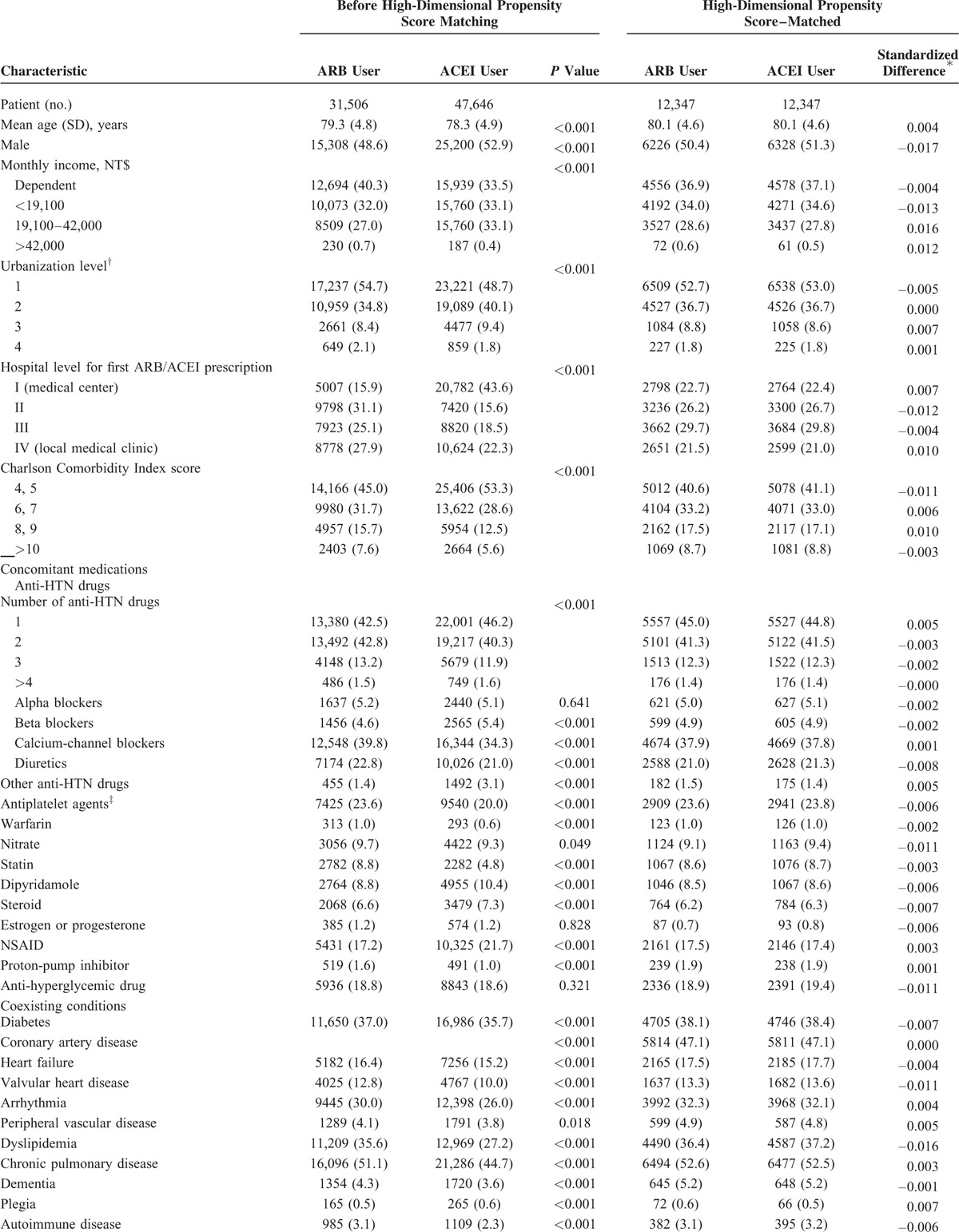
Demographic and Clinical Characteristics of Patients

**TABLE 1 (Continued):**
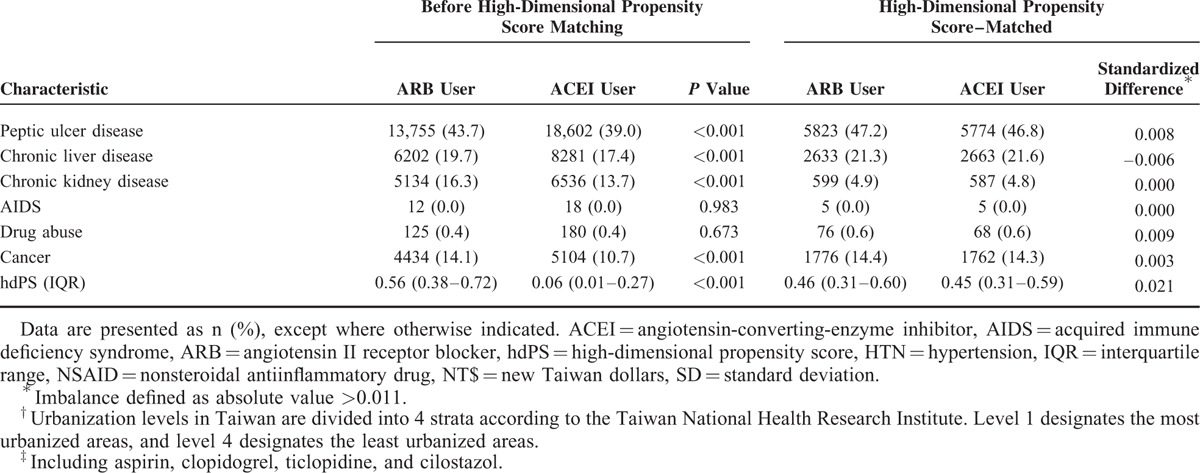
Demographic and Clinical Characteristics of Patients

### ITT Analyses of Long-Term Risks of MACE and Mortality

**FIGURE 1:**
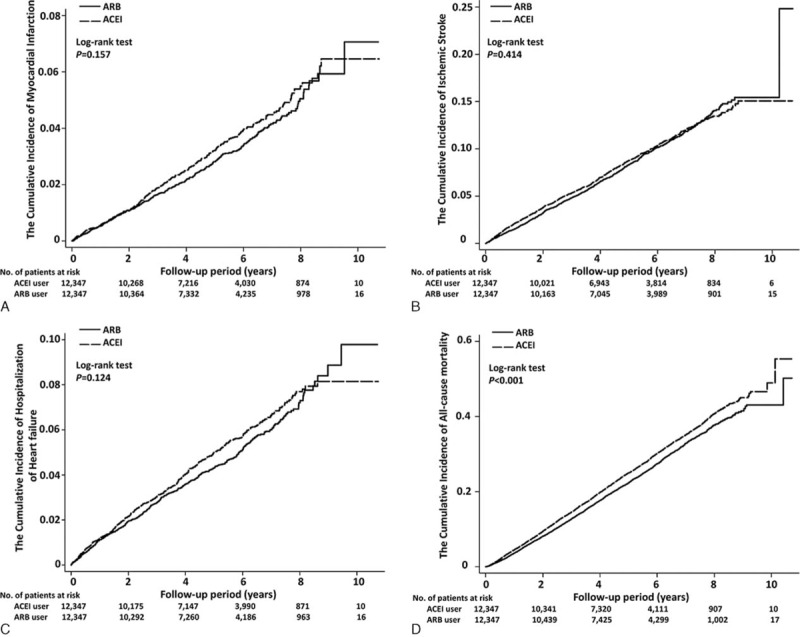
Cumulative incidence of (A) myocardial infarction, (B) ischemic stroke, (C) heart failure, and (D) all-cause mortality in elderly patients using angiotensin II receptor blocker and angiotensin-converting-enzyme inhibitor.

During the mean 6.2-year follow-up period, ITT analyses after hdPS matching showed that ARB use was associated with a lower risk of all-cause mortality (HR 0.89, 95% CI 0.85–0.94; *P* < 0.001; Table 2). After accounting for death as a competing risk factor, ACEI and ARB use had similar effects on the risks of ischemic stroke (HR 0.98, 95% CI 0.90–1.07), myocardial infarction (HR 0.92, 95% CI 0.79–1.06), and heart failure (HR 0.93, 95% CI 0.83–1.04). Regarding adverse effects, the 2 cohorts had similar risks of hospitalization for acute kidney injury (HR 0.99, 95% CI 0.89–1.09) and hyperkalemia (HR 1.02, 95% CI 0.87–1.20). Before matching, MACE risk was also similar in both cohorts after adjustment for hdPS (Supplementary Table 2, http://links.lww.com/MD/A473). Figure 1 illustrates the cumulative incidences of myocardial infarction, ischemic stroke, heart failure, and all-cause mortality in ACEI and ARB users. In subgroup analyses, the treatment effects of ARBs were consistently similar to those of ACEIs (Fig. 2 , Supplementary Tables 4–9, http://links.lww.com/MD/A473).

**TABLE 2:**
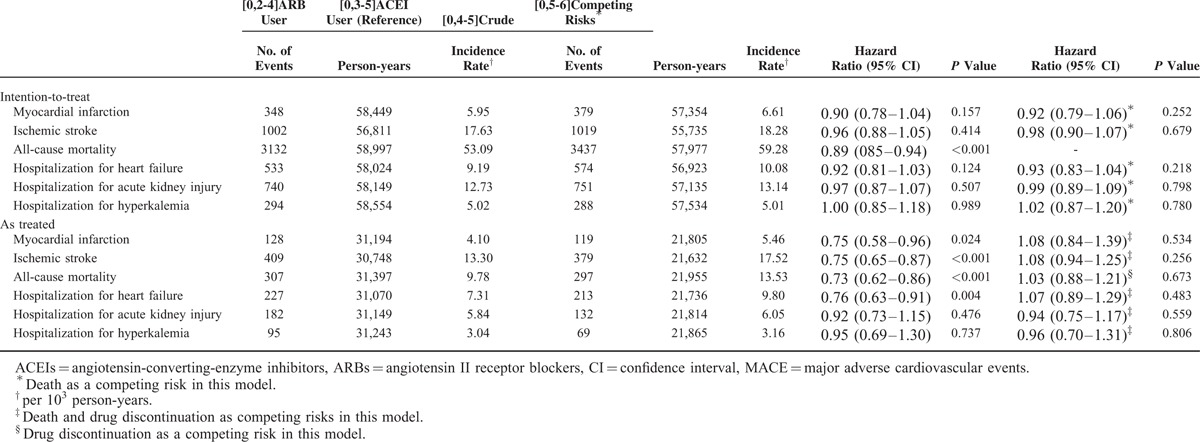
Incidence and Risk of MACE, All-Cause Mortality, and Adverse Effects among Elderly Patients Using ACEIs and ARBs after High-dimensional Propensity Score-Matching

**FIGURE 2:**
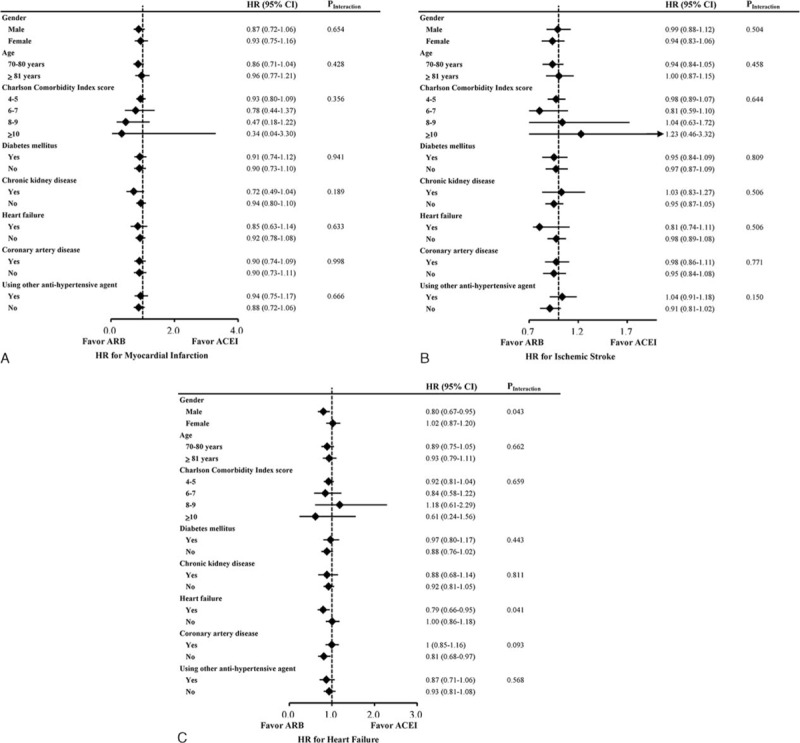
Subgroup analysis of the effects of angiotensin II receptor blocker versus angiotensin-converting-enzyme inhibitor on the risks of (A) myocardial infarction, (B) ischemic stroke, (C) heart failure, (D) all-cause mortality, (E) acute kidney injury, and (F) hyperkalemia in elderly patients.

**FIGURE 2 (Continued):**
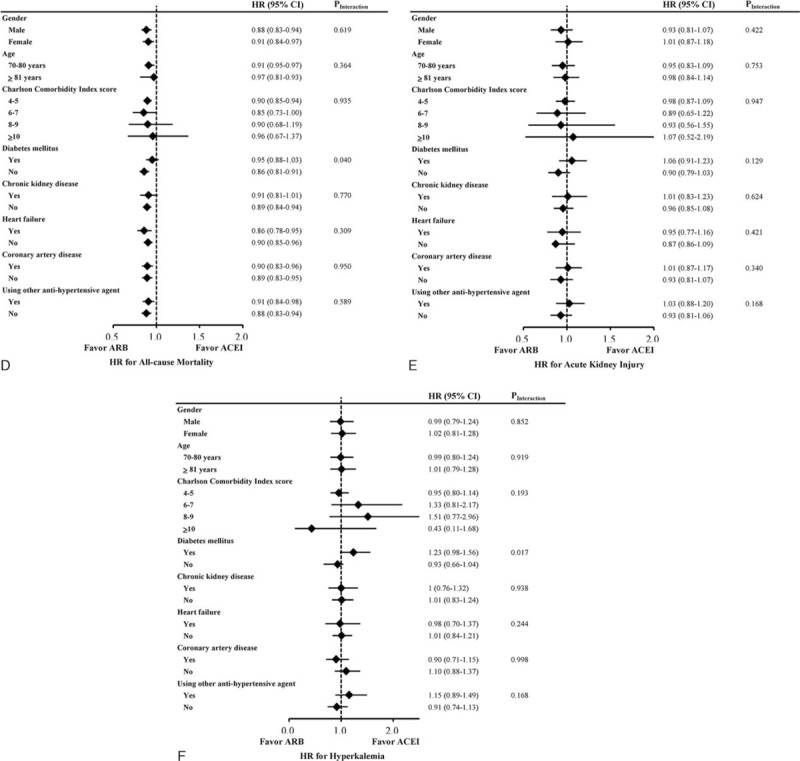
Subgroup analysis of the effects of angiotensin II receptor blocker versus angiotensin-converting-enzyme inhibitor on the risks of (A) myocardial infarction, (B) ischemic stroke, (C) heart failure, (D) all-cause mortality, (E) acute kidney injury, and (F) hyperkalemia in elderly patients.

### AT Analyses of Long-Term Risks of MACE and Mortality

In the AT analysis, the mean durations of continuous drug exposure were 929 (95% CI 915–943) days for ACEIs and 649 (95% CI 638–661) days for ARBs. ARB and ACEI use were associated with similar risks of MACE, acute kidney injury, and hyperkalemia (Table 2, Supplementary Table 3, http://links.lww.com/MD/A473). Of note, after accounting for drug discontinuation as a competing risk factor in AT analysis, the risks of all-cause mortality became similar in both cohorts.

## DISCUSSION

In this hdPS-matched nationwide population-based study, we directly compared the effects of ACEIs and ARBs on the outcomes of MACE and all-cause mortality in a large sample of hypertensive older (aged ≥70 years) patients. ACEIs and ARBs had similar effects on MACE and adverse effects in hypertensive older patients. Subgroup analysis also showed comparative effectiveness of ACEIs and ARBs across stratification subgroups. Although ARBs were associated with a lower risk of all-cause mortality in the ITT analysis, we were unable to demonstrate a difference in the effects of ACEIs and ARBs on the risk of all-cause mortality after considering drug discontinuation as a competing risk in the AT analysis.

Several randomized controlled trials have directly compared ACEIs with ARBs,^17,18,32–34^ but most of these trials have not specifically focused on elderly patients. The exceptions are the ELITE and the ELITE II Study,^17,18^ which compared ACEI and ARB therapy in elderly patients with heart failure. In the initial ELITE study,^17^ losartan-treated patients showed a significant reduction in the risk of all-cause mortality relative to captopril-treated patients; however, in the ELITE II study,^18^ the benefits of ARBs were similar to those of ACEIs. A likely explanation for this difference is that total mortality served as a primary endpoint in the ELITE II study, but as a secondary outcome in the ELITE study. In addition, given the short follow-up periods of these 2 clinical trials, further long-term studies are required.

The results of published observational studies are also inconclusive regarding the comparative effectiveness of ACEIs and ARBs in elderly patients.^19,20^ One hospital-based study of 933 elderly male patients with hypertension found that ACEIs were more effective than ARBs in reducing mortality, as well as cardiovascular and cerebrovascular morbidity. The results presented in that study, however, must be interpreted carefully because of the small sample, inclusion of exclusively male patients, and the cross-sectional study design.^19^ In a cross-sectional study, the outcomes were obtained simultaneously with exposure, and elderly patients who died before the study were also excluded from analysis, which may introduce bias toward including the patients with more favorable survivorship.^35,36^ In contrast to the ELITE II study, a PS-matched observational study of 8049 patients aged ≥ 65 years who had been hospitalized for heart failure showed that ARB use was associated with a lower risk of mortality compared with ACEI use.^20^ However, the results may be limited by the small sample and lack of consideration of drug adherence. Failure to adhere to drug regimens may lead to misclassification bias because subjects who were classified as exposed but were in fact not exposed.^37^

Although ARB use was associated with a lower risk of all-cause mortality compared with ACEI use in the ITT analysis in the current study, the survival benefits of ARB use in elderly patients were offset after accounting for the competing risk of drug discontinuation based on the statistical method used in previous studies.^38,39^ The failure to demonstrate a survival benefit of ACEI use similar to that of ARB use may be explained by lesser adherence to ACEI therapy among elderly patients due to its side effects (eg, cough). Previous studies have consistently found that better adherence leads to better outcomes.^40^ Indeed, the ONgoing Telmisartan Alone and in combination with Ramipril Global Endpoint (ONTARGET) clinical trial directly compared MACE outcomes between ACEI and ARB users. Although this trial did not focus on elderly populations, analysis of treatment effects in the 65 to 74 and ≥75-year subgroups indicated that ACEI and ARB treatments had equal efficacy.^33^

The strengths of our study include the use of a large nationwide population-based dataset from hypertensive patients aged ≥ 70 years. Our follow-up period was longer than those of clinical trials, aiding comparison of the long-term benefits of ACEIs and ARBs in terms of all-cause mortality and MACE in elderly patients. To eliminate the effects of residual confounders that are inherent in observational studies, we used an hdPS algorithm. The ITT analysis retained the original study allocation during the long-term follow-up period, whereas enrollment in the AT analysis ended at the termination of follow-up, drug switching, or drug discontinuation, which might have introduced informative censorship. Because drug discontinuation due to side effects or mortality commonly occurs during follow-up among elderly patients, the use of appropriate statistical methods to consider drug discontinuation and death as competing risks in the analysis is essential.

However, the study findings should be interpreted in the context of several weaknesses. First, data on several potential confounding factors, including body mass index, tobacco and alcohol use, exercise performance, and laboratory tests, were not available and thus were not included in our analyses. Second, the prescription of an ACEI or ARB may be based on the physician's decision between the drugs, which may cause indication bias. However, we performed hdPS matching to control for baseline confounding characteristics in both groups and to reduce the effect of residual confounding.^27,41–43^ Furthermore, Taiwan's NHI provides comprehensive insurance coverage, which could eliminate financial barriers to drug choice when selecting an ACEI or ARB. Third, the database did not contain information on individual blood pressure control. Although the Panel Members Appointed to the Eighth Joint National Committee established the target of 150 mmHg for blood pressure treatment in very elderly individuals based on clinical trial evidence, the benefit of more strict blood pressure control on clinical outcomes in elderly patients remains questionable.^44,45^ In addition, we matched cohorts according to the use of antihypertensive drugs in addition to ACEIs or ARBs, which may partly reflect individual blood pressure control. Fourth, patients aged 65 to 69 years were not included in this cohort. Therefore, the comparative effects of RASS blockade in early elderly (65–74 years) versus late elderly (≥ 75 years) on the long-term outcomes could not be evaluated.^46^ Finally, our study enrolled patients from 2000 to 2009 and followed up until 2010. Therefore, the difference in observed follow-up length of study subjects may bias outcomes. However, we included the index year and index month in our propensity score model in which the follow-up times were more comparable between the ACI and ARB users after matching.

This study bridged evidence gaps regarding the comparative effects of RAAS blockade with ACEIs and ARBs on MACE outcomes in elderly patients. We found that ACEIs and ARBs are equally effective with regard to the risks of MACE and the adverse effects of acute kidney injury and hyperkalemia. When physicians are faced with the choice of using either an ACEI or an ARB in elderly patients, the results of our study provide further evidence to support to take a decision for ACEIs or ARBs based on individualized medical decision and shared decision making.
